# The Goldfish Limbus as a Specialized Epithelial–Stromal Microenvironment: Histological, Immunohistochemical, and Ultrastructural Characterization

**DOI:** 10.3390/vetsci13101004

**Published:** 2026-09-23

**Authors:** Doaa M. Mokhtar, Manal T. Hussein, Eman Fayad, Maha Alsunbul, Mai A. Al-Mosaibih, Fahmy Gad Elsaid, Anthea Miller, Giacomo Zaccone

**Affiliations:** 1 Department of Cell and Tissues, Faculty of Veterinary Medicine, Assiut University, Assiut 71526, Egypt; manal.hussein@aun.edu.eg; 2 Department of Histology and Anatomy, School of Veterinary Medicine, Badr University in Assiut, Assiut 71511, Egypt; 3 Department of Biology, Turabah University College, Taif University, Al-Taif 21974, Saudi Arabia; e.esmail@tu.edu.sa; 4 Department of Pharmaceutical Sciences, College of Pharmacy, Nourah Bint Abdulrahman University, P.O. Box 84428, Riyadh 11671, Saudi Arabia; maalsonbel@pnu.edu.sa; 5 Department of Biological Sciences, College of Science, University of Jeddah, Jeddah 21959, Saudi Arabia; maalmosaibih@uj.edu.sa; 6 Department of Biology, College of Science, King Khalid University, P.O. Box 960, Abha 61421, Saudi Arabia; felsaid@kku.edu.sa; 7 Department for the Promotion of Human Sciences and Quality of Life, San Raffaele University, 00166 Rome, Italy; anthea.miller@uniroma5.it; 8 Department of Veterinary Sciences, University of Messina, 98168 Messina, Italy; gzaccone@unime.it

**Keywords:** limbus, goldfish, CD 117, E-cadherin, telocytes, teleosts

## Abstract

The limbus is a specialized region of the ocular surface, but its structural organization in teleosts remains poorly documented. This study provides the first integrated histological, immunohistochemical, and ultrastructural analysis of the goldfish limbus. The limbal epithelium consisted of stratified non-keratinized epithelial cells supported by a markedly undulating basement membrane and prominent epithelial ridges. The underlying stroma contained collagen fibers, fibroblasts, blood vessels, immune cells, melanocytes, and numerous telocytes. CD117 immunoreactivity was detected mainly in scattered basal and parabasal epithelial cells, with occasional positive stromal cells and telocytes, whereas E-cadherin showed strong membranous expression throughout the epithelium. Ultrastructurally, basal epithelial cells exhibited abundant mitochondria, rough endoplasmic reticulum, and vesicles and were connected by numerous desmosomes and membrane interdigitations. Telocytes displayed elongated moniliform telopodes forming an extensive stromal network, while mast cells, macrophages, and lymphocytes were observed within the epithelial compartment. These findings demonstrate that the goldfish limbus is a structurally complex epithelial–stromal region containing diverse cellular populations. The study provides a morphological framework for future investigations into epithelial maintenance, cellular interactions, and regenerative mechanisms at the teleost ocular surface.

## Simple Summary

The study provides a detailed histological, immunohistochemical, and ultrastructural description of the limbus in goldfish. The limbus showed a specialized epithelial–stromal organization with an undulating basement membrane and diverse epithelial and immune cells. CD117-positive basal cells and stromal telocytes were identified, while E-cadherin showed strong epithelial expression. Ultrastructural examination confirmed well-developed epithelial junctions, immune cells, and characteristic telocyte processes. Overall, the findings provide a useful morphological basis for future studies of epithelial maintenance and regeneration in teleost eyes.

## 1. Introduction

The eye is a highly specialized sensory organ that performs essential functions in feeding, mate recognition, predator avoidance, and, ultimately, the survival and reproduction of vertebrates [1]. The cornea constitutes the transparent anterior surface of the eye and plays a fundamental role in normal vision. Its transparency and optical properties depend on the continuous maintenance and renewal of the corneal epithelium [2]. Corneal epithelial homeostasis is regulated by the proliferation and differentiation of basal epithelial progenitor cells, together with stem/progenitor-cell populations associated with the peripheral cornea and limbal region [3].

The corneal limbus is a specialized transitional zone between the cornea and the surrounding conjunctival and scleral tissues. In addition to serving as an anatomical junction, the limbus provides a specialized cellular microenvironment that supports corneal epithelial homeostasis, regeneration, and repair [4,5]. In mammals, limbal epithelial stem cells (LESCs) generate proliferative progeny that migrate centripetally toward the corneal surface, where they progressively differentiate into mature epithelial cells. This continuous process contributes to the maintenance and repair of the corneal epithelium [6]. Impairment, depletion, or dysfunction of the limbal stem/progenitor-cell population can disrupt epithelial regeneration and, in severe cases, result in corneal opacity and compromised visual function [7].

In addition to its regenerative role, the mammalian limbus performs several important structural and physiological functions. It acts as a protective barrier that restricts the migration and overgrowth of conjunctival epithelium onto the corneal surface. Furthermore, it contributes to the mechanical stability of the cornea and helps maintain its normal curvature and refractive properties. The limbal region is also anatomically associated with the trabecular meshwork, which facilitates aqueous humor drainage and contributes to the regulation of intraocular pressure [8]. The limbal stroma in mammals additionally represents an important immunological microenvironment containing diverse immune-cell populations, including macrophages, mast cells, innate lymphoid cells and soluble signaling molecules. These cells form a complex local immune network that contributes to ocular surface surveillance, tissue homeostasis, and responses to injury or infection [9,10].

Despite the extensive investigation of ocular morphology in fishes, the limbus remains comparatively poorly characterized. Previous studies of teleost ocular tissues have focused mainly on the cornea, including its epithelial organization, stromal architecture, collagen arrangement, and ultrastructural characteristics [11], as well as on the retina and its cellular organization, development, and visual functions [12,13]. Comparative investigations have revealed distinctive structural and ultrastructural characteristics of the teleost cornea and demonstrated evolutionary variation in stromal organization among vertebrates [14]. Similarly, the fish retina has been extensively studied from morphological, developmental, and functional perspectives [15]. 

In zebrafish, several structural and regenerative features of the cornea are shared with those observed in other vertebrates. Nevertheless, the development and organization of the peripheral cornea and corneoscleral limbus remain less well understood than in mammals. During postembryonic development, the zebrafish cornea undergoes progressive maturation characterized by epithelial stratification, cellular differentiation, and remodeling of the peripheral corneal region. These developmental changes suggest that the peripheral cornea and developing limbus constitute specialized regions involved in epithelial maturation and maintenance. However, whether these features correspond to a functionally equivalent limbal stem/progenitor-cell niche to that described in mammals remains unclear [11].

In contrast, comprehensive information concerning the fish limbus is still scarce. Although a limbal region has been anatomically identified at the cornea–sclera junction in certain fish species [16], detailed information regarding its epithelial architecture, stromal cellular composition, immunophenotypic profile, and ultrastructural organization remain limited. For example, a recent anatomical study of Nile tilapia identified the limbus at the cornea–sclera junction but focused primarily on the general organization of the ocular tunics and cornea rather than on the detailed characteristics of the limbal microenvironment [17]. Consequently, the structural organization and potential biological and regenerative significance of the fish limbus remain insufficiently understood. 

Against this background, goldfish (*Carassius auratus*) provides a useful model for investigating the structural organization of the teleost limbus because it is an established experimental species with well-characterized physiological and anatomical features. Goldfish has been extensively used in comparative endocrinology, reproduction, cardiovascular physiology, and other areas of biological and biomedical research [18,19,20]. However, detailed information on the histological, immunohistochemical, and ultrastructural organization of the goldfish limbus remains limited. Therefore, the present study aimed to provide an integrated characterization of the goldfish limbal region, with particular emphasis on its epithelial architecture, CD117 immunoreactivity, immune-cell populations, and stromal telocytes.

## 2. Materials and Methods

### 2.1. Sample Collection

The experimental protocol was reviewed and approved by the Ethics Committee of Assiut University, Egypt (Approval No. 06/2024/0191). Twenty clinically healthy adult male goldfish (*Carassius auratus*) were included in the study. Before sample collection, the fish acclimatized for two weeks under laboratory conditions in aerated holding aquaria containing dechlorinated water. During the acclimatization period, water temperature was maintained at approximately 22–24 °C, pH at 7.0–7.5, and dissolved oxygen at ≥5 mg/L. A 12:12 h light photoperiod was maintained throughout the acclimatization period. The aquaria were continuously aerated, and water quality was monitored regularly to maintain stable environmental conditions. Fish were fed a commercially available goldfish diet once daily, with the amount adjusted to allow complete consumption and avoid excessive accumulation of uneaten feed. These husbandry conditions were maintained consistently throughout the acclimatization period. Fish were considered clinically healthy based on normal swimming and feeding behavior and the absence of visible external abnormalities, including skin or ocular lesions, ulcers, hemorrhages, abnormal pigmentation, fin damage, deformities, or other apparent signs of disease. Only fish meeting these criteria throughout the acclimatization period were included in the experiment. Adult males were selected to provide a relatively homogeneous experimental population and to minimize variation potentially associated with sex and reproductive status.

At the end of the acclimatization period, fish were randomly selected from the holding aquaria. The selected individuals had a mean body weight of 31.50 ± 3.50 g and a standard body length of 8.90 ± 1.10 cm. All selected fish appeared healthy, with no external evidence of disease, trauma, or reproductive activity.

Euthanasia was performed by immersion in an overdose of tricaine methanesulfonate (MS-222; Sigma-Aldrich, St. Louis, MO, USA) at a concentration of 250 mg/L prepared in chilled (4 °C) water. The solution was buffered with sodium bicarbonate to maintain a pH of 7.0–7.2 and prevent acidification [21]. Death was confirmed after complete cessation of opercular movements, followed by an additional observation period of two minutes to ensure the absence of recovery. The euthanasia procedure, exposure time, and confirmation of death were conducted in accordance with the ARRIVE guidelines.

### 2.2. Tissue Sampling and Sectioning

Following euthanasia, both the right and left eyes were immediately enucleated and transferred to a dissection dish containing cold phosphate-buffered saline (PBS). The superior pole of each globe was marked with a microscopic suture to maintain anatomical orientation before the tissue was fixed in 4% paraformaldehyde (PFA) at 4 °C for 16 h. Under a stereomicroscope, the posterior segment, lens, and cornea were carefully separated from the corneoscleral junction using fine micro-scissors, isolating the full circumferential limbal ring. The isolated limbal tissues were then dehydrated through a graded ethanol series, cleared in xylene, and embedded in paraffin blocks for transverse microtomy sectioning.

For histological evaluation, serial sections were prepared from each paraffin block. Five non-adjacent sections from each fish were selected at approximately regular intervals throughout the available limbal tissue to provide representative anatomical sampling. From each selected section, five non-overlapping microscopic fields were examined, resulting in 25 microscopic fields evaluated per fish. Fields were selected systematically according to their anatomical position rather than on the basis of the presence of particular cellular or structural features. A predefined starting field was selected, followed by examination of subsequent non-overlapping fields at regular intervals. Areas containing tissue folds, tearing, processing artifacts, or inadequate tissue orientation were excluded and replaced by the next eligible field.

### 2.3. Immunohistochemistry

For immunohistochemical evaluation, sections representing comparable anatomical levels were examined using the same sampling strategy, and predefined regions of interest were evaluated at a consistent magnification. Immunohistochemical staining was performed using the Pierce™ Peroxidase Detection Kit (Thermo Fisher Scientific, Oxford, UK) according to the manufacturer’s instructions with minor modifications. Paraffin sections were deparaffinized in xylene, rehydrated through graded ethanol solutions, and rinsed in distilled water. Antigen retrieval was carried out by heating the sections in citrate buffer (10 mM, pH 6.0) at 95–98 °C for 10 min, followed by cooling at room temperature for 30 min. The sections were then washed in Tris-buffered saline containing 0.05% Tween-20 (TBST; pH 7.4). Endogenous peroxidase activity was quenched using the kit peroxidase suppressor for 30 min. To minimize background staining, endogenous biotin was blocked with an Avidin/Biotin Blocking Kit (Invitrogen, Waltham, MA, USA), followed by incubation with Universal Blocker™ in TBS for 30 min at room temperature.

The sections were incubated overnight at 4 °C with the following primary antibodies: rabbit polyclonal anti-CD117 (c-Kit) (1:100; Dako, Glostrup, Denmark), and mouse monoclonal anti-E-cadherin (1:100; 13-1900, Thermo Fisher Scientific, Waltham, MA, USA). Negative control sections were processed in parallel by replacing the primary antibody with antibody diluent (Figure S1). The selected antibodies have previously been reported to exhibit cross-reactivity in goldfish tissues and have been successfully applied in fish histological studies [22].

After primary antibody incubation, the sections were washed twice in TBST for 3 min each and incubated for 30 min at room temperature with the appropriate secondary antibodies: goat anti-rabbit IgG (1:1000; 65-6140, Invitrogen, USA) or goat anti-mouse IgG (1:100; 31800, Invitrogen, USA). Following three washes in TBST, the sections were incubated with avidin–horseradish peroxidase (Avidin-HRP; 1:500; 43-4423, Invitrogen, USA) for 30 min. Immunoreactivity was visualized using a metal-enhanced 3,3′-diaminobenzidine (DAB). DAB development was performed for 5–15 min under standardized conditions, with development time kept consistent among sections. Finally, the sections were rinsed, counterstained with Harris’s hematoxylin, dehydrated through ascending grades of ethanol, cleared in xylene, and mounted with DPX mounting medium. For each immunohistochemical marker, the sections were selected from comparable anatomical levels to minimize sampling bias. 

### 2.4. Semithin Sections and Transmission Electron Microscopy (TEM) Preparations 

For ultrastructural examination, limbal tissue samples were collected from the same anatomically corresponding region in each fish. The tissues were immediately fixed in 2.5% glutaraldehyde prepared in 0.1 M phosphate buffer (pH 7.4) [23]. After several washes in the same buffer, specimens were post-fixed in 1% osmium tetroxide for 1 h, dehydrated through a graded ethanol series, and embedded in epoxy resin. Semi-thin sections (0.5–1 µm) were cut using a Reichert Ultracut ultramicrotome (LKB Bromma, Stockholm, Germany), stained with toluidine blue, and examined by light microscopy to identify representative regions for ultrastructural analysis. Ultrathin sections (approximately 70 nm) were then prepared, mounted on copper grids, contrasted with uranyl acetate and lead citrate [24], and examined using a JEOL transmission electron microscope (JEOL Ltd., Tokyo, Japan).

### 2.5. Morphometric Analysis

For morphometric measurements, only well-oriented regions in which the epithelial surface and epithelial–stromal junction could be clearly identified were included. Morphometric analysis was performed on histological sections of the limbus using ImageJ software (v.1.54p., National Institutes of Health, Bethesda, MD, USA). Digital micrographs were acquired at a constant magnification and analyzed after calibration using the scale bar provided in each image.

The following parameters were evaluated: (1) limbal epithelial thickness, measured as the perpendicular distance from the epithelial surface to the epithelial–stromal junction at randomly selected, well-oriented sites; (2) epithelial ridge height, measured from the base of each epithelial ridge at the epithelial–stromal junction to its apical tip along a line perpendicular to the general epithelial surface; and (3) CD117-positive cell density, determined in immunostained sections by counting cells showing distinct brown diaminobenzidine (DAB) immunoreactivity within predefined regions of interest and expressing the results as cells/mm^2^. Diffuse background staining and equivocal staining were excluded from the cell counts.

For each fish, multiple non-overlapping microscopic fields distributed throughout the sampled limbal region were examined for each parameter. Measurements obtained from the individual fields were first averaged within each fish to generate a single animal-level value. Thus, the individual fish, rather than individual sections or microscopic fields, was considered the biological experimental unit, while multiple sections and fields were treated as technical/observational replicates. This approach was used to minimize the risk of pseudoreplication and to account for the dependence of repeated measurements obtained from the same animal.

All morphometric measurements were performed using identical image-analysis settings and magnification throughout the study. Measurements were conducted on well-oriented sections by an observer blinded to specimen identity whenever applicable. The resulting animal-level values were used for descriptive analysis and are presented as mean ± standard error (SE).

## 3. Results

### 3.1. Histological Characteristics of the Goldfish Limbus

Semithin sections revealed a stratified non-keratinized epithelium resting on a markedly undulating basement membrane that projected into the underlying connective tissue as numerous epithelial ridges. The basal epithelial cells possessed a relatively high nuclear-to-cytoplasmic ratio and were closely associated with the folded basement membrane (Figure 1A). In the limbal epithelium, the basal layer was composed of cuboidal to low columnar cells exhibiting large euchromatic nuclei, while the suprabasal layers consisted of polygonal cells with pale cytoplasm and numerous intercellular spaces, whereas the superficial cells appeared flattened and formed the apical surface of the limbus (Figure 1B,C). The morphometric analysis was represented in Table 1. Occasional cells with dendritic-like morphology were identified within the basal and suprabasal epithelial layers. These cells were characterized by irregular cell contours, pale cytoplasm, and elongated or indented nuclei, with slender cytoplasmic processes extending between adjacent epithelial cells. Their cellular profiles differed from the relatively regular polygonal appearance and organized arrangement of the neighboring epithelial cells. Because these cells were identified primarily on morphological grounds and were not characterized using specific cell-type markers, they are referred to here as “dendritic-like cells” rather than assigned to a definitive dendritic-cell lineage (Figure 1A).

Within the basal epithelial layer, occasional large pale epithelial cells with morphological features suggestive of progenitor cells were identified. These cells rested directly on the folded basement membrane and exhibited a lightly stained cytoplasm. Interestingly, some of these basal cells were binucleated, with two closely apposed euchromatic nuclei enclosed within a shared cytoplasm. The apparently binucleated cells, characterized by distinct cellular boundaries and continuous cytoplasm, remained closely associated with the basement membrane and adjacent basal epithelial cells and were sporadically distributed throughout the limbal epithelium (Figure 2A,B). In addition, small lymphocyte-like cells were occasionally observed between the epithelial cells. These cells were characterized by a round to oval profile, a relatively high nuclear-to-cytoplasmic ratio, a densely stained heterochromatic nucleus, and a narrow rim of cytoplasm, and were sporadically distributed throughout the epithelium (Figure 2C,D).

Scattered mast cells containing metachromatic granules were frequently observed among the superficial epithelial layers, particularly toward the conjunctival side of the limbus (Figure 3A). The underlying stroma consisted of loosely arranged collagen bundles, fibroblasts, blood capillaries, and occasional immune cells (Figure 3B–D). Numerous telocytes were distributed throughout the limbal stroma. They exhibited a relatively small cell body with very long, thin cytoplasmic processes (telopodes) extending from the cell body (Figure 3B,D).

### 3.2. Immunohistochemistry

Immunohistochemical examination demonstrated differential expression of CD117 (c-Kit) and E-cadherin within the limbal epithelium. CD117 immunoreactivity was confined mainly to isolated basal and parabasal epithelial cells located immediately above the basement membrane (Figure 4A). These cells exhibited moderate-to-strong cytoplasmic immunostaining, whereas the majority of superficial epithelial cells showed weak or absent labeling. Occasional CD117-positive stromal cells were also detected within the superficial connective tissue beneath the limbal epithelium. Morphologically identified telocytes exhibited CD117 immunoreactivity (Figure 4A). In contrast, E-cadherin exhibited strong and continuous membranous immunoreactivity throughout nearly all epithelial layers (Figure 4B). Immunostaining was particularly intense along the lateral cell membranes of basal and suprabasal epithelial cells, clearly outlining cell boundaries and maintaining epithelial cohesion. Stromal connective tissue cells and extracellular matrix components were largely immunonegative for E-cadherin (Figure 4B). The density of CD117-positive cells was presented in Table 1.

### 3.3. Transmission Electron Microscopy

Transmission electron microscopy provided further evidence of the specialized organization of the limbal epithelium. Basal epithelial cells rested directly upon a continuous basement membrane and possessed large euchromatic nuclei. Their cytoplasm contained abundant free ribosomes, mitochondria, and rough endoplasmic reticulum. Numerous cytoplasmic vesicles were present within differentiating epithelial cells (Figure 5A–D). Adjacent epithelial cells were interconnected by well-developed desmosomes (Figure 5A). The lateral plasma membranes displayed extensive interdigitations that strengthened epithelial cohesion. Basal cells extended slender cytoplasmic processes along the basement membrane (Figure 5C,D). Occasional dendritic-like cells were identified among the basal and suprabasal epithelial cells. Ultrastructurally, they were characterized by an irregularly shaped cell body with cytoplasmic processes extending between adjacent epithelial cells. The nucleus was irregular and predominantly heterochromatic (Figure 5C). Occasional granular leukocyte-like cells were observed between epithelial cells. These cells contained numerous membrane-bound electron-dense cytoplasmic granules of variable size and electron density and exhibited round nuclei with peripheral heterochromatin. Some granules showed lamellar or particulate internal structures, whereas others appeared relatively homogeneous. Based on these ultrastructural characteristics, these cells were classified descriptively as granular leukocyte-like cells (Figure 5C).

Transmission electron microscopy confirmed that the intraepithelial mast cells contained numerous membrane-bound electron-dense secretory granules of variable size and electron density, together with a round nucleus exhibiting peripheral heterochromatin. Some granules displayed lamellar or particulate internal substructures, whereas others appeared homogeneous, reflecting different stages of granule maturation (Figure 6A,B). Occasional intraepithelial macrophages interspersed among the limbal epithelial cells were observed. These cells exhibited an eccentric nucleus. Their cytoplasm contained numerous membrane-bound vesicles of variable size, endocytic vacuoles, lysosome-like electron-dense bodies, and scattered mitochondria. Small phagolysosomal structures and multivesicular bodies were frequently observed. The macrophages lacked desmosomal attachments with neighboring epithelial cells and extended short cytoplasmic processes into the surrounding intercellular spaces (Figure 6A). Binucleated cells were clearly identified in the basal epithelium (Figure 6A). Many intraepithelial lymphocytes could be detected (Figure 6B,C). The basement membrane remained continuous throughout the limbal region and was separated from the stromal connective tissue by a thin lamina densa (Figure 6C).

Telocytes (TCs) exhibited extremely long, thin cytoplasmic prolongations with alternating dilated (podomes) and constricted segments (podomers), forming contacts with neighboring stromal cells and surrounding extracellular matrix. The cytoplasm of these cells contained numerous mitochondria, rough endoplasmic reticulum, and secretory vesicles (Figure 7A–D). Telopodes of TCs connected with each other, forming a network in the stroma (Figure 7C). In addition, occasional melanocytes were also identified within the limbal stroma, where they exhibited dendritic morphology and extended cytoplasmic processes containing melanin granules (Figure 7C).

## 4. Discussion

The present study demonstrates that the goldfish limbus possesses a highly organized epithelial–stromal architecture, although its cellular arrangement differs in several respects from that described in mammalian eyes. The combination of a stratified non-keratinized epithelium, a markedly folded basement membrane, a population of basal CD117-positive cells, strong E-cadherin expression, resident immune cells, and numerous stromal telocytes suggests that the goldfish limbus is a biologically active region rather than simply a passive anatomical junction. These findings characterize the goldfish limbus as a structurally complex epithelial–stromal region. Several of these features resemble components described in regenerative or stem/progenitor-cell-associated microenvironments in other vertebrates; however, the present study provides morphological and immunohistochemical evidence only and does not establish the existence of a functional stem/progenitor-cell niche or a direct role in epithelial regeneration in goldfish. The observed organization therefore provides a structural framework for future investigations of epithelial maintenance and regeneration in the teleost ocular surface.

One of the most conspicuous features of the goldfish limbus was the pronounced undulation of the epithelial–stromal interface. Similar structural specializations of the limbal epithelial–stromal boundary have been described in other vertebrate species and have been proposed to increase the complexity of the stem/progenitor-cell microenvironment [25]. In the mammalian eye, the limbal architecture contains specialized epithelial niches in which epithelial stem/progenitor cells interact with stromal and extracellular-matrix components [26]. These comparative observations provide an anatomical context for interpreting the prominent folding observed in the goldfish limbus; however, they do not establish functional equivalence between the goldfish folds and mammalian stem/progenitor-cell niches. In the present study, the folds were identified as a distinctive morphological feature and were not experimentally assessed for their capacity to increase functional niche area, maintain progenitor cells, regulate epithelial proliferation, or promote regeneration. Therefore, the presence of a folded epithelial–stromal interface should not be interpreted as direct evidence of a stem/progenitor-cell niche in goldfish. At most, the extensive epithelial–stromal interface and close association of basal epithelial cells with the basement membrane provide a structural feature that warrants further investigation. Demonstrating a functional limbal stem/progenitor-cell niche in goldfish will require additional evidence, including validated stem/progenitor-cell markers, proliferation assays, lineage-tracing approaches, and/or functional injury–regeneration experiments.

The basal epithelial compartment deserves particular attention. Ultrastructurally, these cells contained abundant mitochondria, free ribosomes, rough endoplasmic reticulum, Golgi complexes, and cytoplasmic vesicles, indicating considerable metabolic and synthetic activity. Such features are compatible with an actively maintained epithelial compartment. The occasional binucleated basal epithelial cells are also noteworthy. Binucleation by itself cannot be interpreted as evidence of stemness, because it may result from incomplete cytokinesis or altered cell-cycle activity [27]. Nevertheless, the localization of these cells within the basal compartment, their relatively pale cytoplasm, euchromatic nuclei, and close association with the basement membrane make them interesting candidates for future investigation. The present observations therefore support the possibility of a specialized progenitor-associated population but do not establish that these cells are bona fide limbal stem cells. Definitive identification would require additional approaches, including established epithelial stem/progenitor markers, proliferation assays, lineage tracing, or functional regeneration experiments. 

The localization of CD117 provides additional support for the possibility of a specialized basal cell population. In the present study, CD117 immunoreactivity was mainly observed in scattered basal and parabasal epithelial cells, with occasional positive stromal cells and telocytes. CD117/c-Kit has previously been identified among candidate markers associated with limbal epithelial stem/progenitor cells [28]. Human limbal epithelial stem cells have been reported to express c-Kit, together with other surface markers, while c-Kit-positive cells have also been identified in the limbal stromal compartment [29]. Furthermore, c-Kit/SCF signaling has been implicated in interactions between limbal epithelial and niche cells [30]. 

The strong and continuous E-cadherin immunoreactivity throughout the goldfish limbal epithelium provides evidence of well-developed epithelial cohesion. The staining was especially prominent along the lateral membranes of basal and suprabasal cells. This distribution is consistent with the established role of E-cadherin in epithelial cell–cell adhesion and maintenance of epithelial organization [31]. The ultrastructural observations support this interpretation, as neighboring epithelial cells were interconnected by numerous desmosomes and exhibited extensive lateral membrane interdigitations. Together, these findings indicate that the goldfish limbal epithelium possesses substantial mechanical cohesion despite its location at a transitional and potentially dynamic ocular surface. Strong epithelial adhesion may also be important for maintaining the boundary between the limbal epithelium and the underlying stromal compartment while permitting controlled epithelial renewal [32].

Another important feature of the goldfish limbus was the presence of several immune-cell populations within the epithelium and stroma. Mast cells were frequently observed, particularly toward the conjunctival side, while macrophages, lymphocytes, and dendritic-like cells were also identified. The coexistence of epithelial, immune, and stromal cell populations emphasizes that the limbus is a multicellular microenvironment. In mammalian limbal tissues, the stem-cell compartment is likewise influenced by surrounding niche cells and by local interactions between epithelial and stromal components [33]. Thus, the immune cells identified in the goldfish limbus may contribute to local surveillance and tissue homeostasis, although their precise functions remain to be established.

The presence of telocytes within the limbal stroma is another notable finding of this study. Their telopodes extended among collagen fibers and approached neighboring stromal cells, immune cells, and the epithelial–stromal interface. Importantly, telocytes have previously been identified in the mammalian eye, including the limbus and corneal stroma. In the mouse eye, telocytes were observed beneath the limbal epithelium and in close proximity to putative stem cells, while direct contacts and extracellular-vesicle-mediated interactions were suggested as possible mechanisms by which telocytes participate in the stem-cell niche [34]. Telocytes have also been demonstrated in the human corneal stroma where ultrastructural examination revealed long moniliform telopodes and intercellular contacts [35]. More recent work in buffalo has similarly suggested that telocytes and keratocytes may participate in corneal homeostasis and wound repair [36].

The occurrence of CD117-positive telocytes in the goldfish limbus is particularly interesting because it raises the possibility that these stromal cells may be involved in communication with the CD117-positive epithelial population. The close spatial association of telocytes with the basement membrane, collagen fibers, and neighboring cells nevertheless provides a structural basis for proposing that they may contribute to the organization of the limbal microenvironment. Telocytes are increasingly regarded as components of tissue niches that can establish extensive intercellular networks and potentially influence neighboring cells through direct contacts and extracellular vesicles [37]. Therefore, the telocytes identified here may represent an additional stromal component involved in maintaining the structural and cellular organization of the goldfish limbus.

The presence of a continuous basement membrane and close epithelial–stromal association may also provide an important structural scaffold for epithelial maintenance. In mammalian eyes, the extracellular matrix and stromal niche are recognized as important regulators of limbal stem/progenitor-cell behavior rather than merely as passive supporting structures [38]. Taken together, the findings suggest that several cellular components may cooperate to maintain the goldfish limbal epithelium. The basal epithelial compartment contains scattered CD117-positive cells and occasional morphologically unusual binucleated cells; E-cadherin provides strong epithelial cohesion; immune cells occupy both epithelial and stromal compartments; and telocytes form an extensive stromal network close to the epithelial basement membrane. Such organization resembles the general concept of a stem/progenitor-cell niche, in which epithelial progenitors are maintained within a specialized microenvironment rather than functioning independently [39]. In mammals, limbal stem cells are essential for long-term corneal epithelial renewal, and their behavior is closely linked to their surrounding niche [40]. The present observations suggest that comparable principles of epithelial maintenance may exist in goldfish.

A notable finding of the present study was the presence of occasional binucleated basal epithelial cells within the limbal epithelium. Although binucleation alone cannot be regarded as definitive evidence of stemness, their localization within the basal epithelial compartment, their relatively undifferentiated morphology, and their association with scattered CD117-positive cells suggest that these cells may represent a specialized progenitor population. Binucleated epithelial cells have been reported in regenerating tissues of several vertebrates, where they have been associated with incomplete cytokinesis during active proliferation or increased biosynthetic activity [41].

Melanocytes were also identified within the limbal stroma. Although traditionally recognized for their role in melanin production and photoprotection, stromal melanocytes may have additional functions related to local antioxidant defense, immune modulation, and regulation of the tissue microenvironment [42,43,44]. Their occurrence within the goldfish limbus is therefore of particular interest, as they may represent an additional cellular component of the specialized limbal niche. However, the functional significance of these cells in the teleost limbus remains to be determined.

The present study recorded the presence of spaces between the epithelial cells. In teleost fish like goldfish, the ocular surface is anatomically adapted to living entirely submerged in water. Unlike humans, who rely on tight epithelial barriers to prevent the evaporation of tears, freshwater fish face a completely different environment [45]. Goldfish live in a freshwater environment that is hypotonic compared to their bodily fluids. Their ocular surface, especially peripheral regions like the limbus, is constantly managing water and ion exchange. The spaces allow for the controlled movement of fluid and ions to maintain corneal clarity without tearing the tissue apart [45]. Because goldfish do not have eyelids or a blinking reflex, their eyes are exposed directly to the aquatic environment. The flexible spacing allows the epithelium to absorb mechanical micro-shocks from water currents and suspended particles [46]. Furthermore, the limbal region in fish is highly active in housing specialized immune cells macrophages. The intercellular spaces facilitate the migration of these defensive cells through the epithelial layers to quickly respond to aquatic pathogens without causing tissue swelling or inflammation [11].

An important limitation of the present study is that the proposed regenerative significance of the CD117-positive cells and telocytes remains morphological and immunohistochemical rather than functional. In particular, CD117 expression should not be equated with stem-cell identity, and the presence of telocytes does not by itself establish a regenerative function. Future studies should therefore combine CD117 with additional epithelial progenitor/stem-cell markers and proliferation markers and, ideally, employ lineage-tracing or injury-regeneration experiments. Additional characterization of the telocytes using markers such as CD34 and PDGFRα, together with three-dimensional imaging and functional studies, would also help determine whether these cells participate directly in epithelial regeneration or stromal remodeling. Such approaches would be especially valuable in goldfish because teleost tissues frequently display substantial regenerative capacity.

The identification of several intraepithelial and stromal cell populations in the present study was based primarily on their light- and electron-microscopic characteristics. Although their morphology was consistent with particular cellular phenotypes, definitive lineage assignment was not possible in the absence of cell-type-specific immunohistochemical or molecular characterization. Accordingly, cells described as dendritic-like, macrophage-like, or lymphocyte-like should be regarded as morphological classifications rather than definitive lineage assignments. Future studies combining ultrastructural examination with validated cell-specific markers will be required to confirm the identity and functional characteristics of these populations.

## 5. Conclusions

The present study expands the anatomical knowledge of the teleost ocular surface by demonstrating that the goldfish limbus contains a complex epithelial and stromal organization with several features compatible with a regenerative microenvironment. The folded epithelial–stromal interface, basal CD117-positive cells, strong E-cadherin-mediated epithelial adhesion, resident immune-cell populations, and stromal telocytes collectively indicate that the limbus is a structurally specialized region with cellular features potentially associated with epithelial maintenance. The findings provide a morphological framework for future studies addressing epithelial renewal and ocular regeneration in teleosts and may contribute to a better understanding of how regenerative niches are organized across vertebrate species.

## Figures and Tables

**Figure 1:**
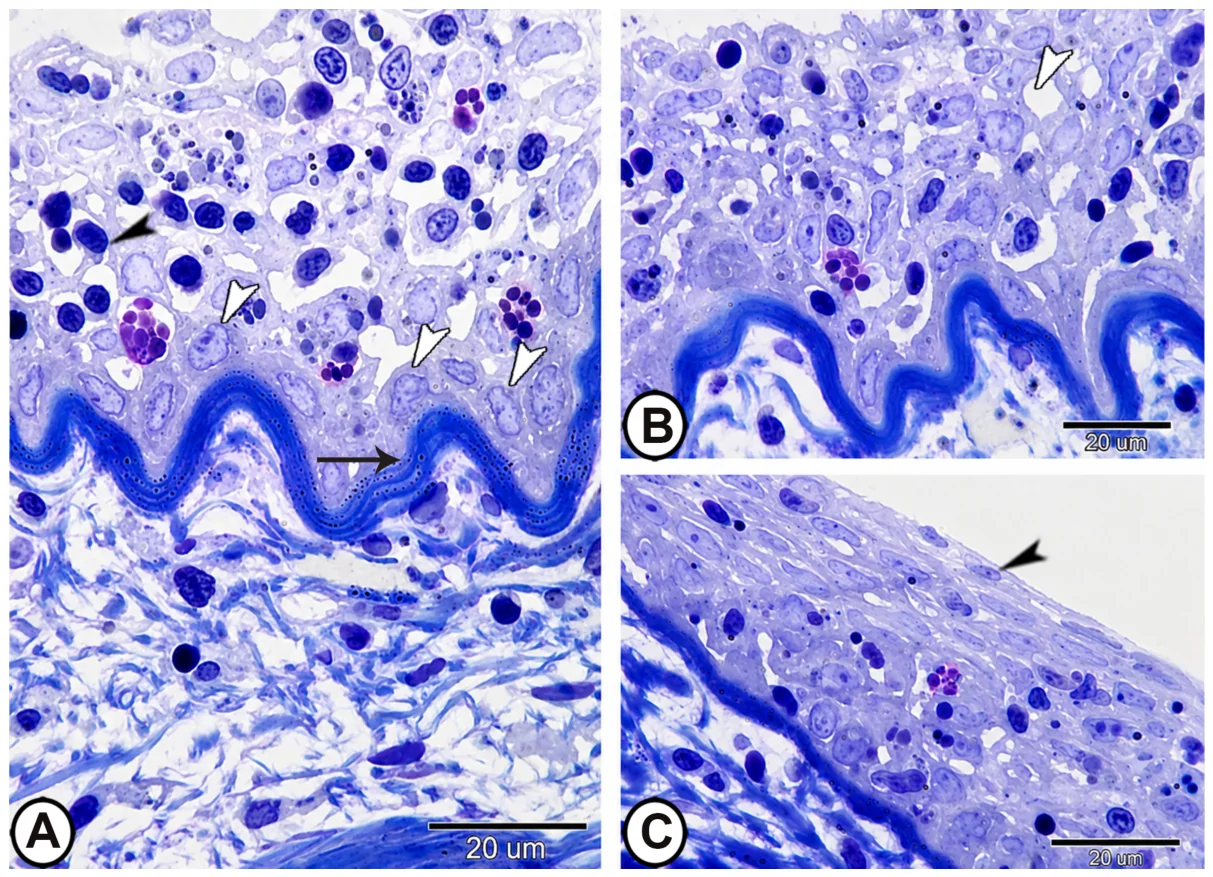
**Semithin sections of the goldfish limbus stained with toluidine blue.** (**A**) The limbal epithelium consists of stratified epithelial cells resting on a conspicuously undulating basement membrane (arrow). Note the dendritic-like cells (black arrowhead) and basal epithelial cells (white arrowheads). (**B**,**C**) The suprabasal layers consist of polygonal cells with numerous intercellular spaces (white arrowhead), whereas the superficial cells are flattened and form the apical surface of the limbus (black arrowhead).

**Figure 2:**
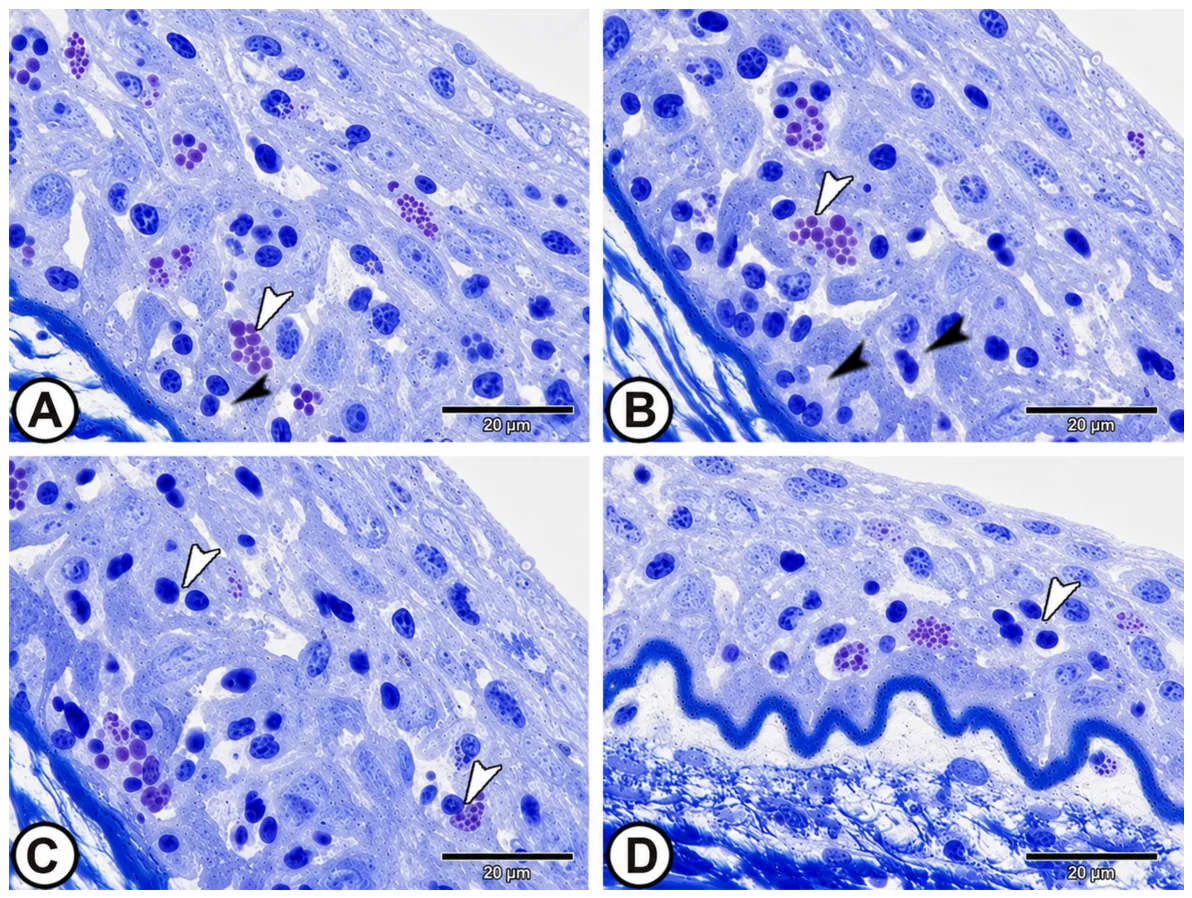
**Semithin sections of the limbal epithelium stained with toluidine blue.** (**A**,**B**) The epithelium contains multiple resident cell populations. Numerous mast cells (white arrowheads) containing metachromatic cytoplasmic granules are distributed among epithelial cells. Binucleated basal cells (black arrowheads) are also identified adjacent to the basement membrane. (**C**,**D**) Small lymphocyte-like cells (arrowheads) were sporadically encountered between epithelial cells.

**Figure 3:**
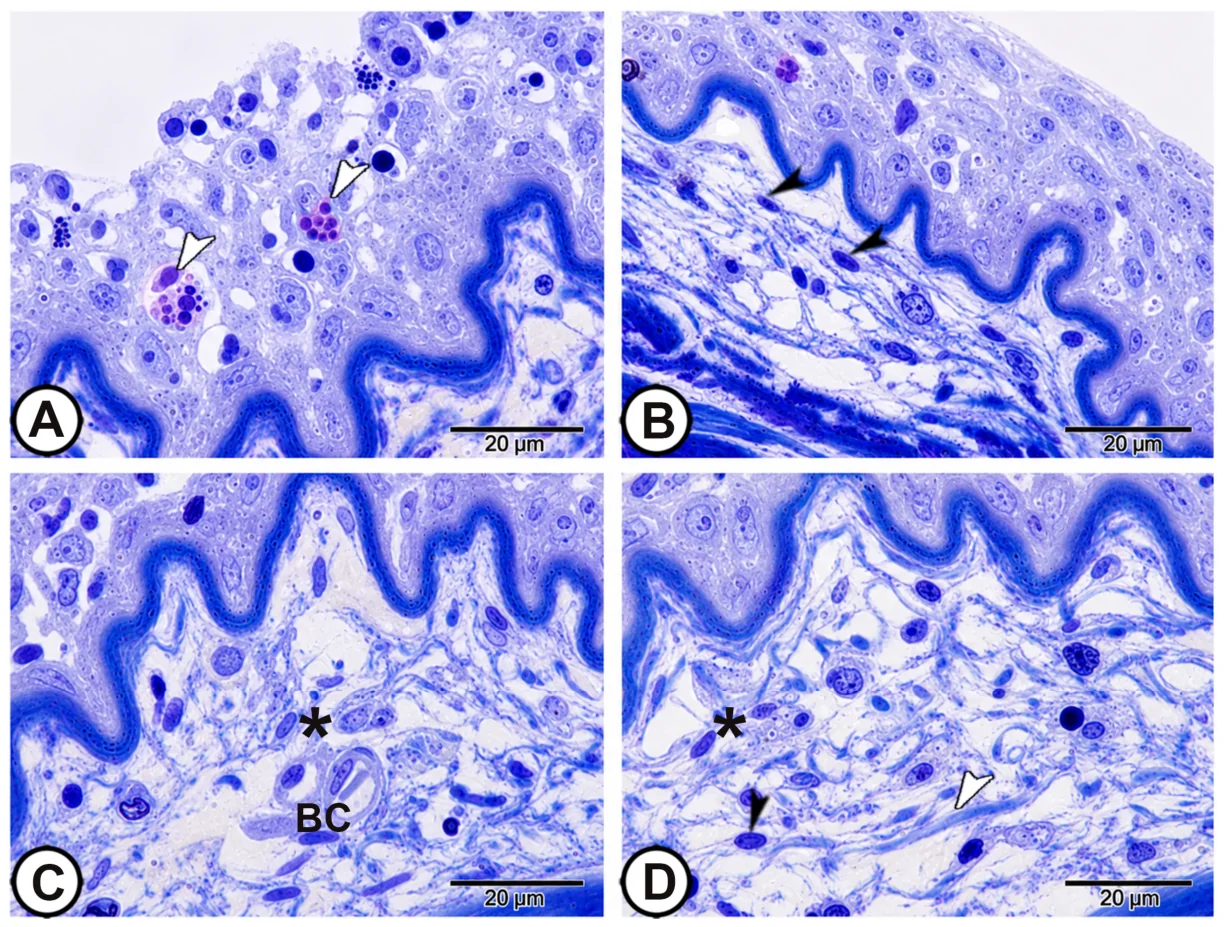
**Semithin sections of the limbal stroma stained with toluidine blue.** (**A**) Scattered mast cells (arrowheads) were frequently observed among the superficial epithelial layers. (**B**–**D**) The underlying stroma consisted of loosely arranged collagen bundles (white arrowhead), fibroblasts (asterisks), blood capillaries (BC), and numerous telocytes (black arrowheads) distributed throughout the limbal stroma.

**Figure 4:**
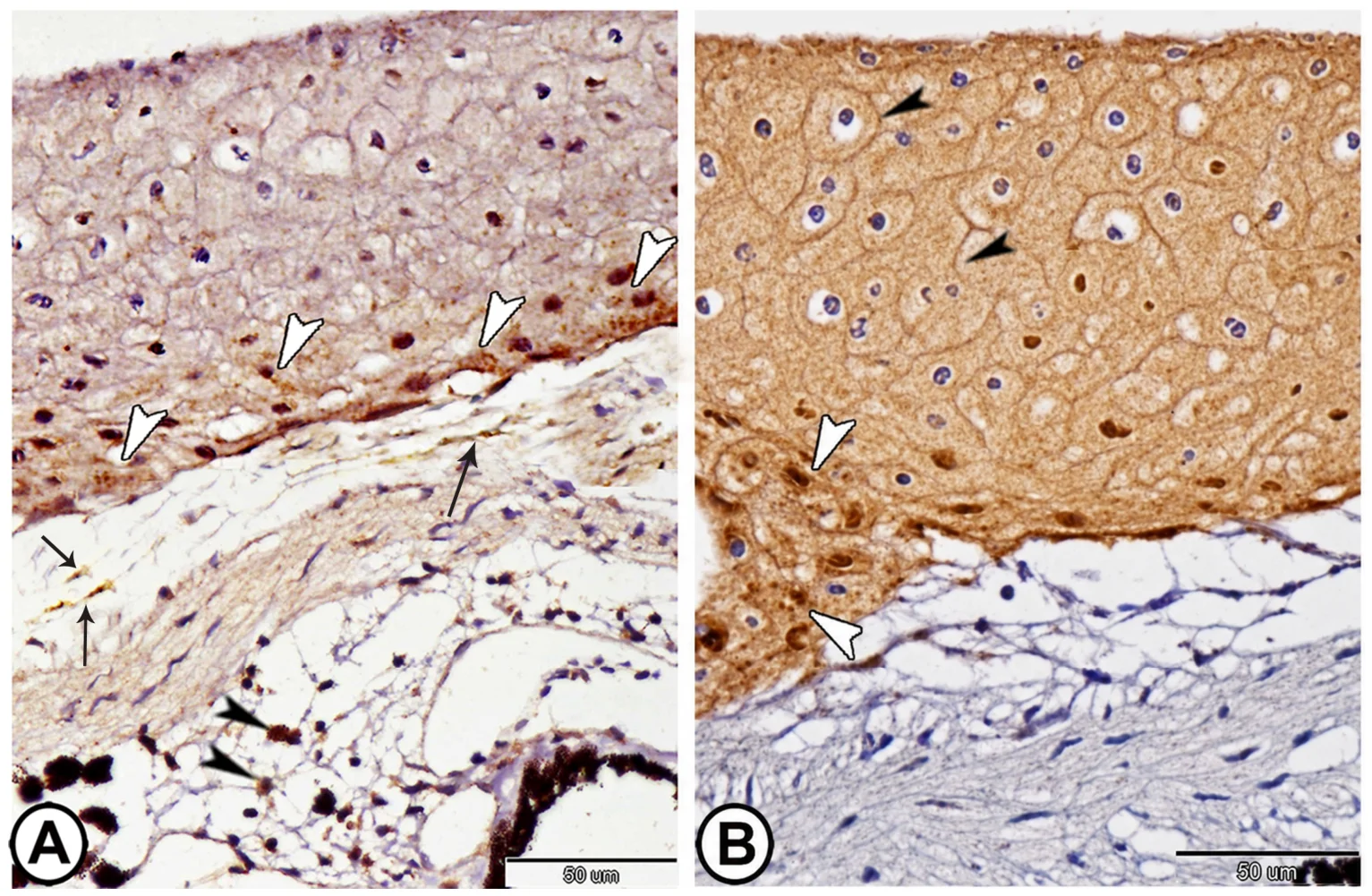
**Immunohistochemical localization of CD117 (c-Kit) and E-cadherin in the goldfish limbus.** (**A**) Moderate-to-strong cytoplasmic CD117-immunoreactivites are observed in basal epithelial cells (white arrowheads) and some stromal cells (black arrowheads). Telocytes (arrows) in the stroma also expressed CD117. (**B**) Strong membranous E-cadherin immunoreactivity is observed along the lateral borders of epithelial cells (black arrowheads). The staining is particularly prominent within the basal and suprabasal epithelial layers (white arrowheads).

**Figure 5:**
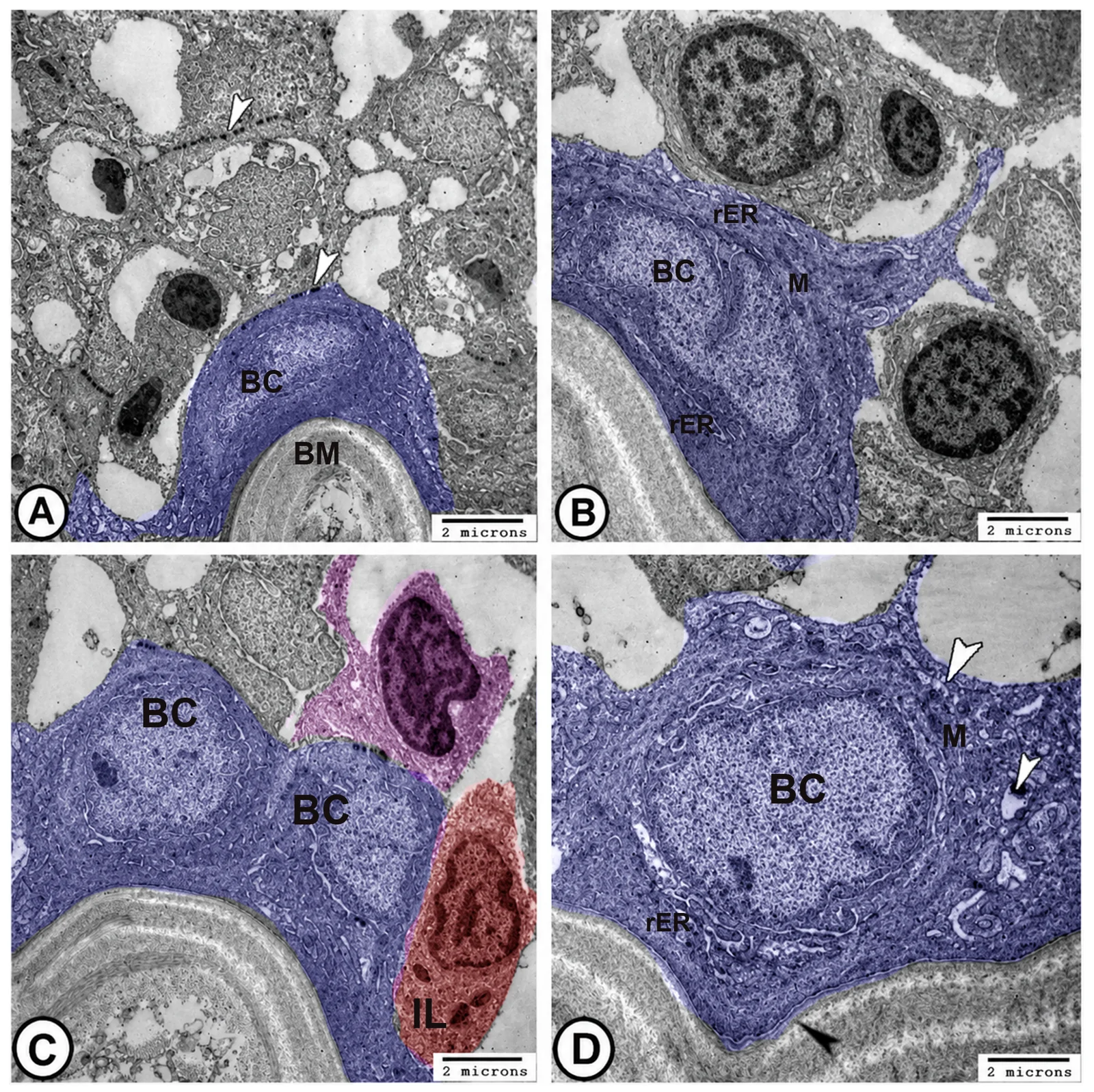
**Digital colored TEM images show the limbal epithelium.** (**A**,**B**) Basal epithelial cells (BC, blue) rested directly upon a continuous basement membrane (BM). Their cytoplasm contained abundant rough endoplasmic reticulum (rER) and mitochondria (M). Adjacent epithelial cells were interconnected by desmosomes (arrowheads). (**C**,**D**) Basal cells extended slender cytoplasmic processes along the basement membrane (black arrowhead). The cytoplasm of basal cells contains numerous cytoplasmic vesicles (white arrowheads), rER, and mitochondria (M). Occasional dendritic-like cells (pink) and intraepithelial granular leucocyte-like cells (IL, red) were identified among the basal and suprabasal epithelial cells.

**Figure 6:**
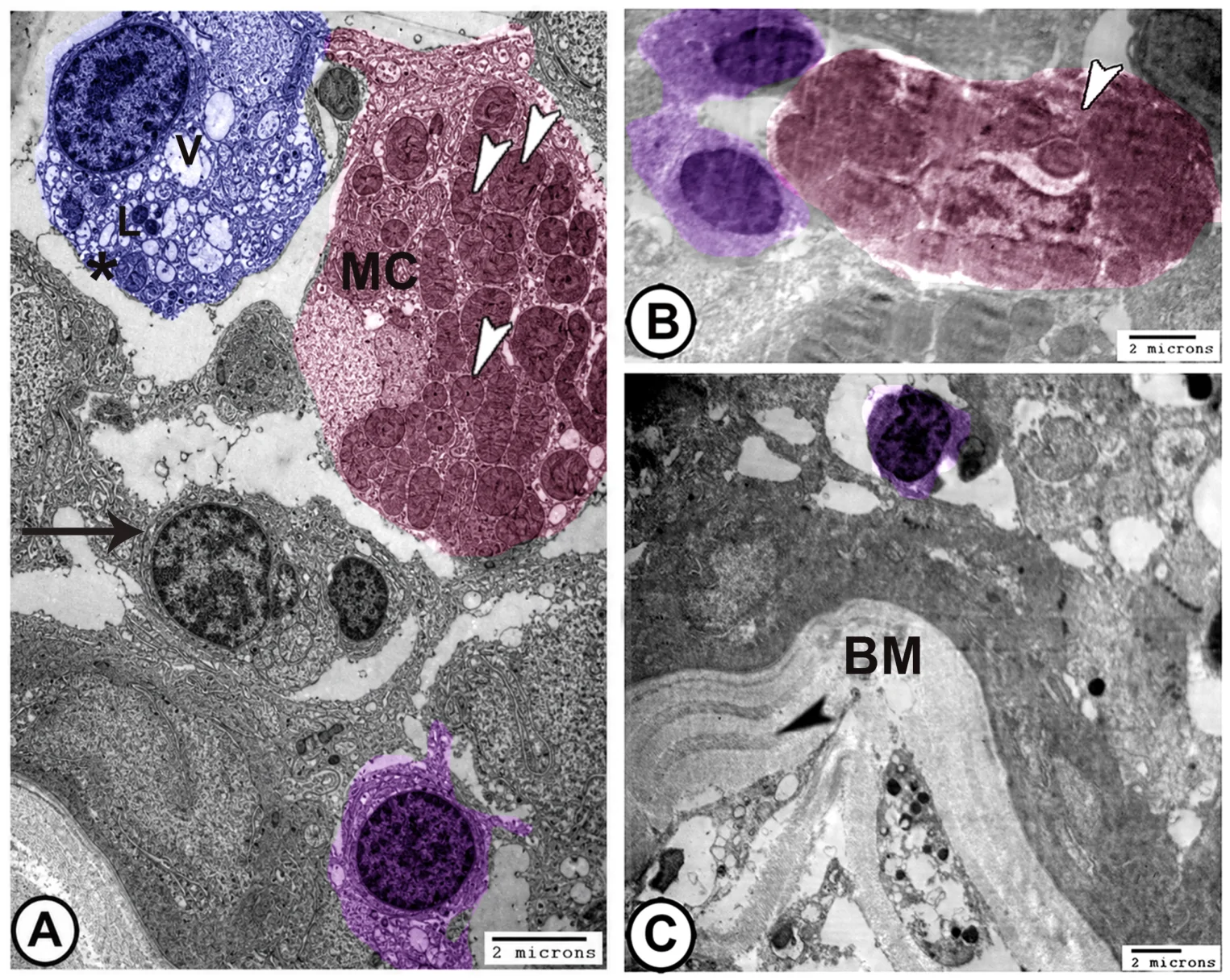
**Digital colored TEM images showing the immune cells in the limbal epithelium.** (**A**) An intraepithelial macrophage-like cell (blue) interspersed among epithelial cells. Its cytoplasm contains abundant endocytic vesicles (V), lysosomes (L), and phagolysosomes (asterisk). Mature mast cells (MC, pink) containing numerous electron-dense secretory granules (arrowheads). Note the intraepithelial lymphocyte-like cell (violet) and the binucleated cell (arrow). (**B**,**C**) Many intraepithelial lymphocyte-like cells could be detected (violet). Mast cells (pink) with granules (white arrowheads). The basement membrane is separated from the stromal connective tissue by a thin lamina densa (black arrowhead).

**Figure 7:**
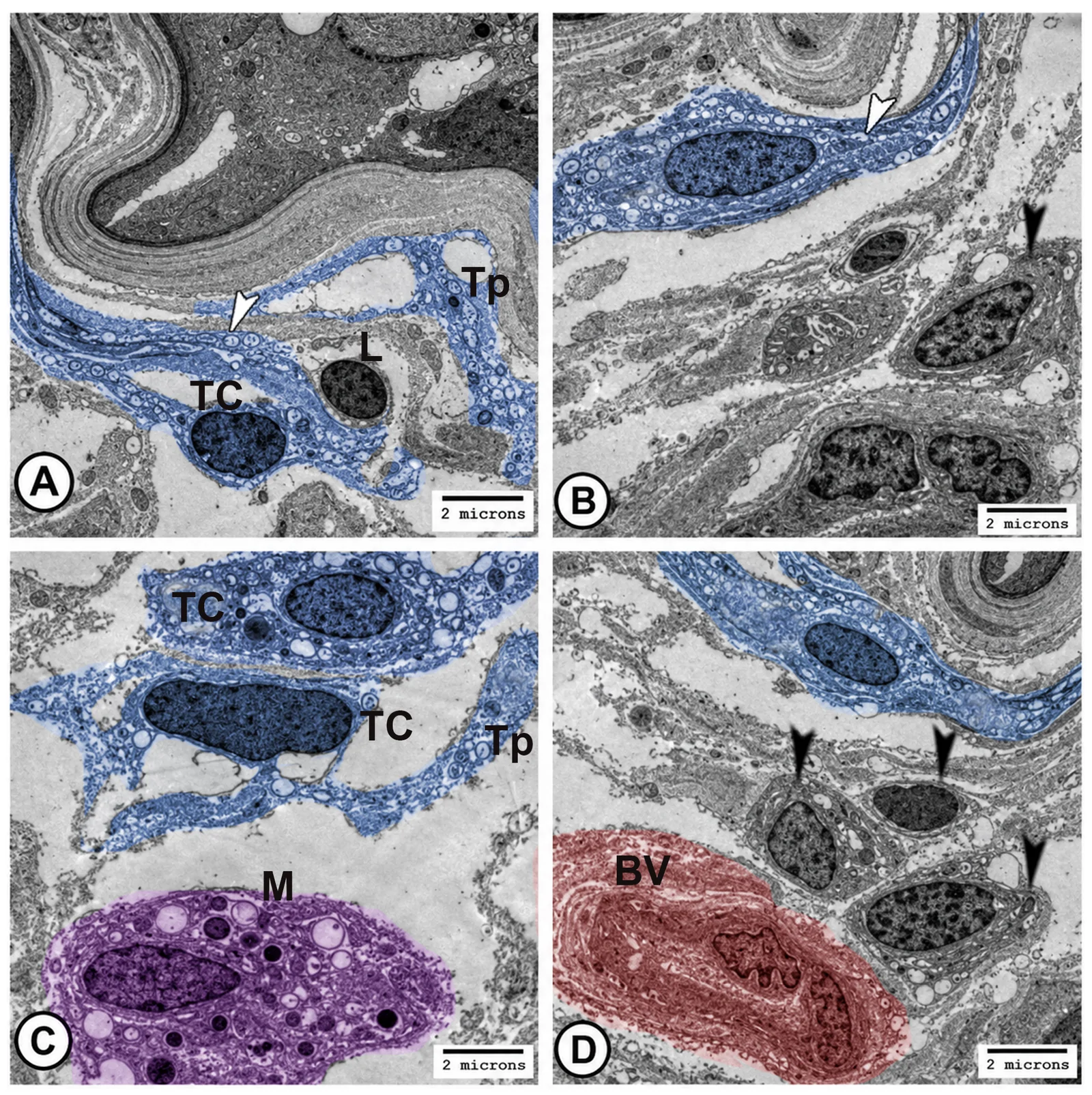
**Digital colored TEM images showing telocytes within the limbal stroma.** (**A**–**D**) Telocyte-like cells (blue, TC) with elongated telopodes (Tp) exhibiting alternating podoms and podomers and containing secretory vesicles (white arrowheads). Telopodes extend among adjacent immune cells (black arrowheads). Blood vessels (BV, red) and melanocytes (M, violet) containing melanin granules are also evident within the stroma. L indicates a lymphocyte-like cell.

**Table 1:** Morphometric Analysis of the Goldfish Limbus.

Parameters	Value	Unit
Epithelial thickness	39.2 ± 2.1	µm
Ridge height	26.4 ± 3.7	µm
CD117-positive cell density	135 ± 9.0	cells/mm^2^

## Data Availability

The original contributions presented in this study are included in the article/Supplementary Material. Further inquiries can be directed to the corresponding author.

## Supplementary Materials

The following supporting information can be downloaded at https://www.mdpi.com/article/10.3390/vetsci13101004/s1. Figure S1: Negative controls for E-cadherin immunostaining performed on goldfish limbus sections processed without primary antibodies.
