# Supplementary material for: The Goldfish Limbus as a Specialized Epithelial–Stromal Microenvironment: Histological, Immunohistochemical, and Ultrastructural Characterization
Source: Vet Sci. 2026 Sep 23;13(10):1004. doi: 10.3390/vetsci13101004 (PMC13709404; doi:10.3390/vetsci13101004)

Figure S1. Negative controls for E-cadherin immunostaining performed on goldfish limbus sections processed without primary antibodies. No specific immunoreactivity was detected.

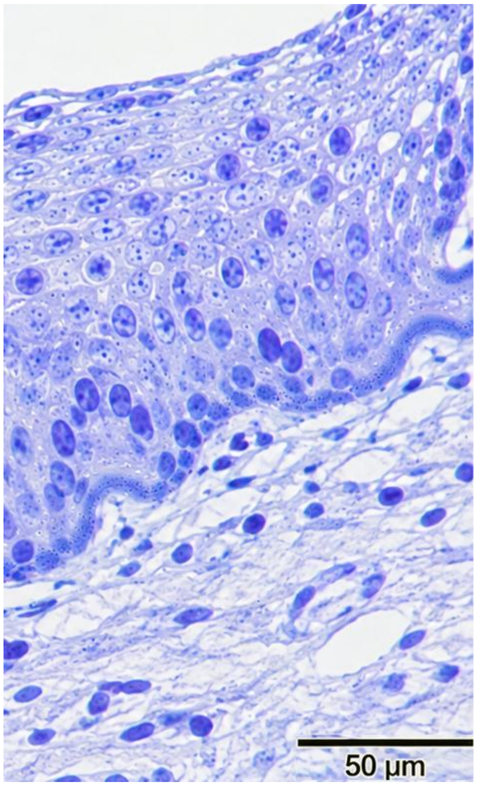

Supplement: Supplementary file 1 [file vetsci-13-01004-s001.zip › vetsci-4551215-supplementary.pdf]
